# Causal manipulation of feed-forward and recurrent processing differentially affects measures of consciousness

**DOI:** 10.1093/nc/niaa015

**Published:** 2020-09-07

**Authors:** Christopher Allen, Tommaso Viola, Elizabeth Irvine, Jemma Sedgmond, Heidi Castle, Richard Gray, Christopher D Chambers

**Affiliations:** n1 Cardiff University Brain Research Imaging Centre (CUBRIC), School of Psychology, Cardiff University, Maindy Road, Cardiff, CF24 4HQ, UK; n2 Institute of Neuroscience, Medical School, University of Newcastle, Newcastle upon Tyne, NE2 4HH, UK; n3 Philosophy, School of English, Communication and Philosophy, John Percival Building, Cardiff University, Colum Road, Cardiff, CF10 3EU, UK

**Keywords:** transcranial magnetic stimulation, blindsight, conscious perception, unconscious processing, signal detection theory, feed-forward/recurrent

## Abstract

It has been theorized that cortical feed-forward and recurrent neural activity support unconscious and conscious cognitive processes, respectively. Here we causally tested this proposition by applying event-related transcranial magnetic stimulation (TMS) at early and late times relative to visual stimuli, together with a pulse designed to suppress conscious detection. Consistent with pre-registered hypotheses, early TMS affected residual, reportedly ‘unseen’ capacity. However, conscious perception also appeared critically dependent upon feed-forward processing to a greater extent than the later recurrent phase. Additional exploratory analyses suggested that these early effects dissociated from top-down criterion measures, which were most affected by later TMS. These findings are inconsistent with a simple dichotomy where feed-forward and recurrent processes correspond to unconscious and conscious mechanisms. Instead, different components of awareness may correspond to different phases of cortical dynamics in which initial processing is broadly perceptual whereas later recurrent processing might relate to decision to report.

**Highlights**

- Disruption of feed-forward activity affected conscious *and* unconscious processing more than later interference.
- Exploratory analysis indicated later interference changes response criteria.
- Feed-forward/recurrent processing might better reflect perception/report than unconsciousness/consciousness.

## Introduction

**Figure 1.**
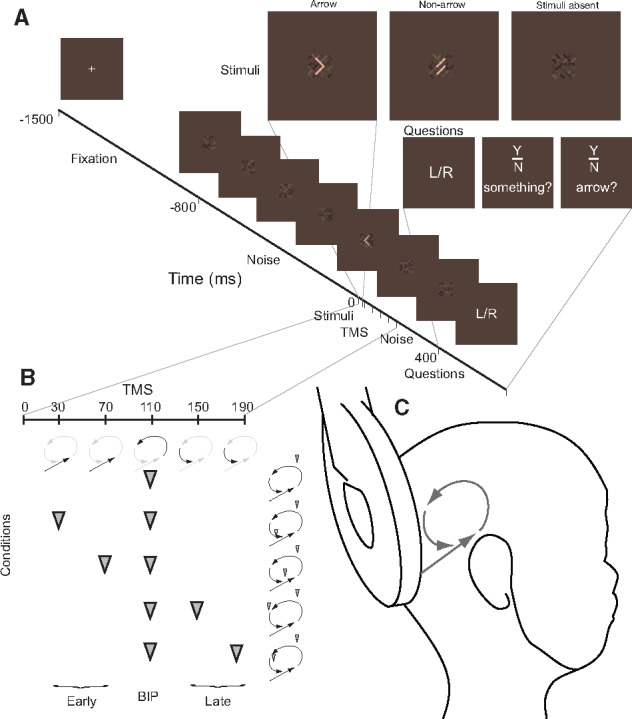
Experimental design. (**A**) Depicts the time course of a single trial. The three stimuli conditions are presented, including stimulus present (arrow), non-arrow and stimulus absent (noise frame). Questions are as presented to participants at the end of each trial in counterbalanced order. Two primary measures traced (i) participants conscious awareness of the stimuli (PrC) via their response to the ‘did you see’ arrow and something yes/no questions and (ii) a measure of residual ‘unseen’ capacity (PcU) which was based on left/right performance capacity when participants reported not having seen the arrow or something (see Materials and Methods section and participant instructions). (**B**) Illustrates the different TMS conditions, indicating the interventions applied at the period termed the Blindsight Inducing Pulse, BIP (110 ms), together with separate pulses on the same trial during an ‘early’ phase to target feed-forward processing (30 or 70 ms), or during a ‘late’ phase to interfere with later, putatively recurrent processing (150 or 190 ms). (**C**) Illustrates the approximate positioning and orientation of the 90-mm round TMS coil used in the experiment and a cartoon representation of feed-forward and recurrent processing

One of the most influential functional descriptions of how and when consciousness manifests in the human brain is the suggestion that the initial feed-forward and later recurrent sweeps of activity support unconscious and conscious processing, respectively (Lamme and Roelfsema 2000; Lamme *et al.* 2000; Lamme 2001, 2006a). Correlational evidence links early electrophysiological visual components to reportedly ‘unseen’ masked stimuli (Fahrenfort *et al.* 2007). However, direct causal evidence, involving a manipulation of ‘unseen’ capacity, is lacking though there have been unsuccessful attempts to manipulate it (Koivisto *et al.* 2011; Koenig and Ro 2019). Disruption of cortical activity very early on, relative to stimuli presentation, has not been shown to interfere with unconscious perception, as would be expected under Lamme’s theory. Conscious awareness of stimuli, by contrast, not only correlates with the presence of later field potential activity (Lamme *et al.* 1999) but interference with relatively late (>∼100 ms) visual cortical activity using transcranial magnetic stimulation (TMS) has repeatedly been shown to suppress conscious awareness (Amassian *et al.* 1989; Allen *et al.* 2014). Where such a suppression dissociates from preserved ‘unseen’ perceptual capacity, regardless of TMS onset time, it is known as TMS-induced blindsight (Ro *et al.* 2004; Boyer *et al.* 2005; Jolij and Lamme 2005; Allen *et al.* 2014), which can also be interpreted as a form of unconscious perception. The aim of the current experiment was to determine the relationship between feed-forward/recurrent activity and conscious/unconscious processing using TMS to differentially interfere with early and later processing (see Fig. 1). This involved applying TMS at different times, relative to the presentation of stimuli, where TMS applied early disrupted feed-forward processing to a greater extent than when TMS was applied at later times.

In addition to being suggested as critical for conscious processing in general, recurrent activity has been predicted more specifically to be important for so-called ‘phenomenal’ consciousness (see Block 2005; 2007, page 498), but to date this proposal lacks empirical support. Although notoriously difficult to define, phenomenal consciousness is associated with the ‘what it is like’ experiential aspect of consciousness (Block 2011). Phenomenal consciousness is supposed to be (potentially) distinct from access and reportability. The most commonly cited examples used in support of phenomenal consciousness are paradigms based on the work of Sperling (1960), in which participants report having been consciously aware of display items (e.g. an array of letters) the content of which they cannot access or report (precisely which letters were displayed) unless cued. This has been interpreted as evidence of participants having been phenomenally conscious of a rich scene, but where working memory constraints limits the amount they can access and report at any one time. The measures in our experiment were designed to track the same response types as found in the Sperling task, and in particular the case where participants acknowledge conscious phenomenal awareness of stimuli but fail to perform a forced choice discrimination associated with it, indicating a lack of working memory capacity or access (see Materials and Methods section). By measuring the proportion of such responses during TMS-induced disruption of recurrent processing, we sought to put Block’s (2007) prediction to the test.

Our study thus tested three pre-registered hypotheses. First, consistent with previous observations (Ro *et al.* 2004; Boyer *et al.* 2005; Jolij and Lamme 2005; Allen *et al.* 2014), our baseline hypothesis was that occipital TMS applied at 110 ms post-stimulus onset should suppress conscious perception while residual, reportedly ‘unseen’ perceptual discrimination would remain above chance. Our primary experimental hypothesis was that an additional TMS pulse applied during the early feed-forward sweep should disrupt ‘unseen’ discrimination sensitivity to a greater extent when compared with later TMS. Finally, our secondary hypothesis was that an additional TMS pulse applied during later processing should disrupt ‘phenomenal’ consciousness to a greater extent than earlier TMS.

## Materials and Methods

### Overview

The methods described here match those pre-registered (https://osf.io/d7uik/), where any deviations, aside from tense and clarifications, have been made explicit. All procedures received ethical approval from the Cardiff University, School of Psychology ethics committee in accordance with the declaration of Helsinki. Data, analysis programmes and copies of stimulus materials are available at https://osf.io/dwfqv/.

### Participants

Seventy-nine participants attended an initial session all of whom had passed initial TMS safety screening (Maizey *et al.* 2013). Fifty participants (22.4 mean age ±3.2 SD range 19–37, 34 female) went on to complete the full experiment which involved between 8 and 12 h of testing over 3–8 sessions separate by at least 24 h. Of the 79 participants who initially signed up for the experiment, 13 did not continue to the main experiment owing to inability to perceive phosphenes within safety limits, a further 4 participants did not take part owning to large or uncomfortable facial twitches, 12 withdrew voluntarily (see Exclusion criteria section).

### Design

One common strategy to probe consciousness is to contrast measures that track conscious awareness of stimuli against perceptual measures where awareness is lacking in some way – with the difference between them revealing what might be involved in consciousness (Lau and Passingham 2006). Blindsight, in various forms, is an archetypical example of this dissociation. Our paradigm was designed to measure conscious and unconscious perception and be capable of demonstrating dissociations between these measures. Arrow target stimuli were presented against luminance noise on half of all trials (Fig. 1A). Of the remaining trials, half (25% of total) contained a non-arrow stimulus and half contained only noise (stimuli absent) (Fig. 1A). Following each presentation subjects were asked three questions: ‘Did you consciously see the arrow? (Yes or No)’, ‘Did you see something? (Yes or No)’ and ‘Was it pointing Left or Right? (Fig. 1A). Participants were instructed to respond positively to the ‘arrow’ question if they are aware of the arrow and to respond positively to the ‘something’ question if they were aware of either the arrow or the non-arrow being present. Additionally, they were instructed to use the ‘something?’ question to indicate any awareness they felt they had of the stimuli possibly being present. In this way, the ‘something?’ question was designed to reflect a lower level of awareness than the ‘arrow?’ question. With respect to the left/right discrimination, participants were also instructed to offer their best guess as to the direction of the arrow, irrespective of whether or not they believed the arrow is present. Responses were unspeeded. Full task instructions can be found in Supplementary Materials and were available to participants in each session. In addition to pre-registered procedures, verbal instructions were regularly repeated to participants, especially if the participant expressed some confusion with respect to making a directional decision on the basis of what they believed to be an absence of information, as was often encountered. The content and structure of these instructions were largely maintained in order to minimize variability between experimenters who collected data (C.A., J.S., T.V., H.C.), where participants were told ‘If you see it say you saw it, if you don’t say “no” and always try your best on the left/right decision even if you don’t think anything was presented’.

Previous research has shown that applying TMS at around 110 ms post-stimulus onset is effective in producing TMS-induced suppression awareness (Amassian *et al.* 1989) and blindsight (Boyer *et al.* 2005; Allen *et al.* 2014). The primary intervention of this experiment was the application of TMS at this time and to combine this with the application of additional single pulses on the same trials either before or after this pulse, termed the ‘Blindsight Inducing Pulse’ (BIP). The term BIP is used for consistency with the pre-registered protocol. However, as it transpired, the data did not indicate a complete dissociation between conscious perception and reportedly ‘unseen’ discrimination, so should not be interpreted as producing blindsight (see Results section). The BIP was experimentally necessary as previous research (Allen 2012; Allen *et al.* 2014; Koenig and Ro 2019) and piloting indicated that single pulse TMS, or pairs of TMS pulses in rapid succession, are largely ineffective in changing residual ‘Unseen’ discrimination. The BIP therefore enabled us to probe the primary experimental hypotheses. As the BIP interferes with later recurrent processing, and also because all TMS effects only carry forward in time, the differences between temporal intervention conditions are *relative* ones, in which early feed-forward processing is disrupted to a greater extent when TMS is additionally applied before the BIP, in comparison to when it is applied after the BIP. The times chosen for these additional pulses were at 30, 70, 150 and 190 ms from stimulus onset (see Fig. 1B). This meant that there were five temporal TMS conditions, one where the BIP was applied in isolation to demonstrate a TMS-induced suppression of awareness while residual ‘unseen’, potentially above-chance, discrimination might be demonstrated, and four in which pairs of pulses were applied at the BIP and additional times designed to target different stages of processing. When TMS was applied at the BIP (110 ms) and at 30 or 70 ms, the data relatively probed the role of the early feed-forward sweep, whereas TMS applied at the BIP (110 ms) and at 150 and 190 ms informed the role of recurrent processing. The use of two time points before and two time points after the BIP was intended to increase the likelihood of affecting processing. It also allowed for the examination of the time course of any disruption with greater accuracy than would be possible if only one additional TMS pulse was administered before or after the BIP. These finer granularity time points were only examined when appreciable differences between early versus late interventions were observed.

Each participant completed 12 blocks of active TMS and 12 blocks of control (sham) TMS, each consisting of 80 trials. Each participant therefore completed 1920 experimental trials. As there were four stimulus conditions (left arrow, right arrow, non-arrow and stimulus absent) and five temporal TMS conditions (BIP in isolation, 30ms&BIP, 70ms&BIP, BIP&150ms and BIP&190ms), each block contained four repetitions of each unique condition, the order of which was randomized. The number of blocks completed on any single day of data collection varied according to factors such as participants’ availability.

The control condition was sham stimulation in which the coil is placed over the target region but oriented such that the magnetic flux entering the scalp is minimal. Blinding to this control was attempted; although participants are often aware of the differences between active and sham TMS. Since the central questions of this investigation involved contrasting two active TMS conditions (early vs. late effects), this temporal control makes the issue of ineffective blinding less problematic than is the case with the majority of sham controlled experiments. Due to the large area affected by a round TMS coil (Roth *et al.* 1991), and the possible involvement of a wide range of areas in the processes under investigation, vertex or other site-based controls were not suitable.

There were six possible orders in which the experimental questions could be presented, all of which were used over the course of the experiment. A single order of questions was maintained throughout each block and participants completed four consecutive blocks of the same question order before a new order was applied. The order in which the six question orders were applied was randomized for each participant. When each new question order was introduced, participants were given 10 practice trials to familiarize themselves with the task. These practice trials were not analysed. An equal number of active and control blocks were completed for each question order, resulting in six possible active/control orders per question order. Each participant completed a full set of active/control orders and these were randomized for each participant independently of question order.

### Measures

Non-parametric signal detection theory was applied to derive a measure of conscious detection. Table 1 describes the conscious detection measure (PrC) in terms of signal detection theory classes (Macmillan *et al.* 1990; Corwin 1994). This is the non-parametric equivalent of detection dʹ. The measure of conscious detection also takes account of reflective reports made by participants following their participation in an almost identical task (Allen *et al.* 2014). That is, the allocation of response patterns to signal detection categories was informed by reports made by participants about their experience of the task (Varela 1996). For example, responding negatively to the ‘arrow?’ question and positively to the ‘something?’ question in the presence of a non-arrow was classed as a ‘correct rejection’, as opposed to a ‘hit’, because participants felt the primary way they approached the task was the detection of the arrows, not detection of the non-arrows. Reportedly ‘unseen’ sensitivity (PcU) was quantified by Left/Right discrimination capacity when participants respond negatively to both the ‘arrow?’ and ‘something?’ questions (Cheesman and Merikle 1987; Dienes 2004). This measure was therefore composed of trials where participants had twice reported the stimuli as ‘unseen’ and where residual perceptual sensitivity was indicated by left/right performance being greater than chance at 50% (PcU > 0.5).

**Table 1.** Signal detection theory classes for measures of conscious awareness (PrC)

	Response		
Stimulus	Something?	Arrow?	SDT Class
Arrow	Yes	Yes	HIT
	No	No	MISS
Non-arrow	Yes	Yes	FA
	Yes	No	CR
Nothing	Yes	Yes	FA
	Yes	No	FA
	No	No	CR

FA, false alarms; CR, correct rejections; hit rate = hits/(hits + miss); false alarm rate = FA/(FA+CR); PrC = hit rate – false alarm rate (Corwin 1994).

A central aim of this experiment was to assess if and how reportedly ‘unseen’ discrimination capacity was affected by TMS applied at different times. However, if a participant were to have demonstrated no evidence of residual ‘unseen’ capacity in baseline conditions, then the central question would have been unanswerable when applied to their data. Therefore, an amendment was made, early in the data collection (https://osf.io/x9pig/), to apply additional criteria in which participants were excluded from the analysis involving PcU, if they did not demonstrate evidence of baseline (sham) ‘unseen’ capacity. Individual PcU performance in the sham condition (across TMS time conditions) was assessed relative to chance using a one-tailed z-test based upon a cumulative normal distribution, where chance is PcU = 0.5, the number of trials contributing to PcU measures was the ‘n’ coefficient and alpha was 0.05. If a participant’s ‘unseen’ performance was not greater than chance, then they were excluded from the analysis of PcU only. This resulted in the removal of eight participants.

The primary hypothesis was tested by the analyses of changes in the PcU and PrC measures. To probe the secondary hypothesis an additional measure (PCm) was developed, to reflect the presence of ‘phenomenal’ consciousness relative to conscious access. The intention here was not to question Block’s proposed conceptual distinction, but rather to probe an empirically tractable prediction made by Block with respect to phenomenal consciousness. Block appeals to several conditions which express phenomenal consciousness in isolation, but the example most often cited in recent works are the experiments conducted by Sperling (e.g. Sperling 1960, cited in Block 2007, 2011). In these experiments, participants are shown an array of (typically 12) letters for a brief period of time, of which they acknowledge awareness (they state that they saw an array of letters). Participants can generally only freely identify 3–4 letters out of each display. However, given a post-stimulus cue to any of the rows in the display, subjects can identify the letters in that row. According to Block, this suggests that participants are phenomenally conscious of all the specific letters in the array, but cannot access information about all of them at any one time, due to working memory constraints. A specific prediction made by Block is that this behavioural manifestation of phenomenal conscious will be accompanied by recurrent processing (Block 2007, page 498). If this is so, then the current experiment is a valid way of testing whether recurrent processing is associated with phenomenal consciousness through the demonstration of expressions of phenomenal consciousness and the manipulation of recurrent processing.

The PCm was designed to follow the similarities between the current experimental configuration and the interpretation of the Sperling experiments by Block (2007, 2011), using detection without identification as an appropriate measure of phenomenal consciousness in contrast and relative to conscious access [which Block (2012), still seems to support]. The numerator of the PCm was acknowledged awareness, indicated by positive responses to both detection questions, while the directional discrimination judgement was made incorrectly and the denominator was all arrow present trials, see Supplementary Method for further details.

In addition to the primary measures (PrC and PcU), the same Signal Detection Theory (SDT) classes could be used to drive a measure of bias (BrC) which was applied as an exploratory measure, where BrC = False Alarm Rate/(1-PrC) (Corwin 1994). This is the bias component of the primary conscious detection sensitivity measure (PrC).

Following a reviewer’s suggestion, it may be informative to quantify sensitivity and criteria to the ‘Arrow?’ and ‘Something?’ questions separately. The allocation of the signal detection classes for these measures is described in Supplementary Table S2. Pr (sensitivity) and Br (criteria) measures (PrA, PrS, BrA and BrS where A stands for Arrow and S stands for Something) were derived through the application of non-parametric signal detection theory and identical analyses were applied as with all other exploratory measures.

**Table 2.** Summary of temporal order comparisons applied to measures

	Measure	T	*p*	df	mean	95% CI		*d*	BF_main_	BF_main_	BF_uni_	BF_uni_	BF_jzs_	Number
									_early>late_	_late>early_	_early>late_	_late>early_		outliers
Primary	PcU	−1.93	0.06	40	−0.03	−0.07	0.00	−0.30	**3.82**	0.19	1.20	0.03	0.90	1
	PrC	−4.17	0.00	48	−0.06	−0.08	−0.03	−0.60	1734.41	**0.05**	408.90	0.01	187.88	0

Secondary	PCmA	0.33	0.74	47	0.00	0.00	0.01	0.05	0.64	0.91	NA	NA	0.17	1

Exploratory	BrC	−2.16	0.04	46	−0.03	−0.06	0.00	−0.32	3.59	0.34	NA	NA	1.31	2
	PrA	−2.96	0.00	46	−0.04	−0.06	−0.06	−0.43	29.96	0.11	NA	NA	7.12	2
	BrA	−0.73	0.47	46	−0.01	−0.04	−0.04	−0.11	1.14	0.83	NA	NA	0.20	2
	PrS	−4.12	0.00	47	−0.04	−0.06	−0.06	−0.59	1415.17	0.05	NA	NA	156.92	1
	BrS	−1.42	0.16	47	−0.03	−0.06	−0.06	−0.20	1.61	0.17	NA	NA	0.40	1

Highlighted in bold are the predicted effects critical to the interpretation of the primary hypotheses. *d* is Cohen's d and 95% CI are the 95% confidence intervals around the mean difference. BF refers to Bayes Factor, where ‘main’ applies a prior based on half the mean difference between the active and sham conditions. ‘Uni’ refers to a uniform prior, when applied to PcU its range is equal to the range from chance to its peak value irrespective of TMS, for PrC its range is 0.5. Jzs refers to the default prior described by (Rouder *et al*., 2009) scaled to 0.707.

### Calibration

Prior to the experimental sessions, participants attended a threshold session where, following a period of familiarization with the task, the stimuli were calibrated by adjusting the luminance of the target to produce a PrC level of 0.5 used in the subsequent experimental session. This involved generating a psychophysical function of each participant’s conscious detection performance over a range of stimuli luminance intensities, which could then be solved for a PrC level of 0.5. Each data point in this function was the result of performance over a mini block consisting of 20 trials. Once the participants’ specific luminance was established, a number of additional mini blocks were completed to ensure stability. If a fluctuation in PrC performance was observed beyond a criterion level of ±0.1 PrC units then small adjustments were made according to the psychophysical function such that performance was maintained at a PrC of ≈0.5. At the start of each experimental session, participants also completed a mini training block and similar adjustments were made as required. This session-specific updating of baseline stimulus levels was designed to lessen the impact of day-to-day variation in task performance. Each active TMS block was accompanied by a sham block in which the luminance of the targets was equal. When more than two blocks were completed within a single experimental session the participants’ performance over the control block served as equivalent to a mini block and so indicated whether an adjustment of the stimuli should be made for subsequent blocks.

During the threshold session, participants were also screened for suitability for TMS (see Maizey *et al.* 2013) and a phosphene threshold (PT) obtained (Franca *et al.* 2006). The method used here resembled that of Franca *et al.*, (2006) and has been previously described (Allen et al. 2014, https://osf.io/d7uik/).

### Equipment

TMS was administered with a Magstim high-power 90-mm round coil in conjunction with a Magstim Rapid^2^ biphasic stimulator. Pulse delivery was controlled via a Cambridge Research Systems (CRS) Visage running Real Time Sequencer software on a Matlab platform, which also governed stimulus presentation on a gamma-corrected 21” Mitsubishi CRT monitor (100 Hz vertical refresh rate) at a viewing distance of 720 mm. To expand upon the stimuli details described in the pre-registration document: each trial commenced with a 1500 ms fixation period, followed by the onset of the luminance noise, consisting of each bar in the matrix (see Fig. 1) pseudo-randomly alternating in luminance (range 25 candela/m^2^, white noise distribution) every 20 ms. After 800 ms the stimuli or a noise frame (stimulus absent) appeared for 20 ms as an addition to the noise of a uniform increase in luminance, the level of which was determined according to the participants calibration. This followed by a further 400 ms of noise before the questions were displayed. The noise occupied 1.9° of visual angle and the stimuli subtended to 0.8° × 1.4°.

TMS pulses in the experimental sessions were delivered at 120% of participants PT. If participants expressed discomfort during the threshold session when TMS was applied at 120% of PT the TMS intensity was reduced to 110% of PT. Three participants received stimulation at 110% of PT. The mean level of absolute stimulation intensity as a proportion of maximum stimulator output was 78.0% ± 13.7 SD. It is possible, as with almost all TMS experiments involving online suppression of visual capacity, that phosphenes could mask the stimuli. However, the time course of the widely reported effects of TMS applied to the visual cortex is difficult to reconcile with the notion of the phosphene, rather than the simpler interpretation that the TMS itself, acts as a mask. Furthermore, even if phosphene were to mask the stimuli, this should not dramatically alter the interpretation of the experiment.

The TMS coil was oriented with the handle pointing upwards and side ‘B’ facing the participant, so that the induced current passed initially in a left-to-right direction. Coil positioning was initially based on anatomical proximity to the calcarine sulcus, localized in individual structural Magnetic Resonance Imaging scans. Immediately prior to each TMS block, the intensity was set to 130% of PT and the coil was moved so that it produced a phosphene that the participant reported as being ‘reasonably clear’ and, ‘at least in part, covering the centre of their visual field’ with their eyes closed. The coil position was then recorded using a Brainsight system (Rogue Research Inc.) and used for the subsequent block of trials. If the participant was observed to have moved beyond a 5 mm tolerance of the original coil position, indicated by the brainsight system, then the block was paused and the coil repositioned to the recorded site. An approximation of this position was used in the sham condition but with the coil perpendicular to the scalp so that the rim pointed towards the head, and with a 10.6-mm plastic spacer inserted between the scalp and coil to replicate the contact artefact.

To exclude effects of TMS-induced blinks on performance, eye tracking was undertaken throughout the experiment using a CRS chin-rest-mounted infrared eye tracker (250 Hz). Trials were excluded on the basis of blinks identified by a shift in pupil position (>1°) followed by a transitory (<1 s) loss of pupil signal, coincident with the stimulus presentation. This resulted in the exclusion of 205 out of 96 000 trials. One block for one participant was excluded from the pupillometry analyses due to a data saving error.

### Statistical analyses

The first question posed in the analysis was whether the BIP in isolation produced a blindsight-type effect. This was to be demonstrated by a significant suppression of conscious detection during active TMS relative to sham, while concurrent ‘unseen’ performance was above chance and ideally maintained. A paired *t*-test was used to compare active to sham PrC performance, and a one sample *t*-test was used to compare PcU performance to chance (PcU≈0.5) in the active condition. A series of corresponding Bayesian analyses were also implemented (see below). PcU performance was also tested for the effects of TMS using a paired *t*-test. If PcU performance over the BIP was suppressed by the TMS, in addition to PrC, then this lessened the extent to which the observed phenomena was classified as a form of blindsight because the dissociation between the two measures (PcU and PrC) would be incomplete. However, such a demonstration did not negate the ability of the experiment to demonstrate other temporally bound effects of the TMS, such as the temporal dynamics of unconscious vision.

The main analysis of this experiment involved the PcU measure: comparing the effects of TMS applied before versus after the BIP. Using the sham-normalized data, a paired *t*-test compared data collected when TMS was applied before the BIP (PcU Δ sham mean of 30ms&BIP and 70ms&BIP) to when it was applied afterward (PcU Δ sham mean of BIP&150ms and BIP&190ms).

A number of additional analyses were implemented to investigate the effects of the TMS timing on measures with the finer resolution (e.g. comparing the effects of 30ms&BIP to the effects of 190ms&BIP). These analyses were only to be implemented if the primary analyses, described above, warranted their application through the demonstration of reliable effects (*P* < 0.05 or B >3 or <1/3, see below).

For all proposed paired *t*-tests, corresponding Bayesian analyses were implemented (Dienes 2008, 2011, 2014; Gallistel 2009; Rouder *et al.* 2009). As described (https://osf.io/x9pig/) measures were assessed relative to a sham TMS baseline and concerned a comparison between the temporal TMS conditions. Small changes were therefore more probable than larger changes within a potential range of fluctuations from sham. Bayesian tests applied to temporal comparisons were based upon half normal priors, starting at zero, whose variance was equal to half of the mean difference between the orthogonal active/sham conditions. Bayesian tests using such priors are relatively assumption free and therefore conservative, defaults. This was the main test applied to temporal order effects and the resultant Bayes Factor is described as BF_main_.

As the PrC measures had a maximum range of 0.5 (from threshold level of 0.5 to 0) this was used as the upper limit for prior uniform distributions representing the current hypothesis (see Dienes 2008, 2014). The range of the prior representing any changes to PcU spanned from the peak value, irrespective of TMS conditions, to chance levels at 50% correct and the resultant Bayes Factors are denoted as BF_uni_. In addition to pre-registered tests we applied a Bayesian test with a default JZS prior (BF_jzs_) as has become standard practice (Rouder *et al.* 2009). This applied a default scaling factor of 0.707 in line with standard recommendations but representing the expectation of a large/medium effect size in all applications. These tests were applicable to both change from sham and temporal comparisons tests. With respect to the temporal comparisons, owing to the double baselining (active vs. sham and early vs. late) and consequent reduction in effect size, it is noted that these are conservative tests.

By implementing these Bayesian statistics we were justified in continuing to collect data until either substantial support for the hypothesis was obtained (BF_main_ ≥ 3) or, conversely, substantial support for the null was obtained (BF_main _≤ 1/3, Jeffreys 1961; Dienes 2011). Therefore, only when either of these conditions were satisfied (B >3 or <1/3), for the primary analysis of PcU comparing early and late effects, conventional *t*-tests were implemented, reported and data collection terminated (July 2018). Where comparisons of active to sham TMS were required, the vector representing the effect for each dependent measure was active minus sham, and a hypothesized suppression was represented by a negative prior. If comparison between early and late effects was required and the prediction was for an earlier suppression then the vector was as follows: (active 30ms&BIP minus sham 30ms&BIP, active 70ms&BIP minus sham 70ms&BIP) minus (active BIP&150ms minus sham BIP&150ms, active BIP&190ms minus sham BIP&190ms), which was integrated with a negative prior. If the hypothesis was for a later effect, then this same vector was integrated with a positive prior. Additionally, priors with the opposing directionality were implemented to assess potential unpredicted effects (see Supplementary Table S3).

In terms of phenomenal consciousness, the main question was whether or not the measure of phenomenal consciousness (PCm) was suppressed when TMS was applied relatively late compared to earlier applications. The same structure of tests applied to the PcU measure was also appropriate for this PCm analysis. However, as the PCm measure was, as far as we are aware, completely novel, its range and variance could not be easily predicted *a priori* although the half normal approach described above was applicable. Because it is possible that the BIP in isolation may have been sufficient to disrupt recurrent processing and therefore suppress phenomenal consciousness, an active versus sham set of analyses was implemented comparing active BIP versus sham BIP and active versus sham where data were collapsed across temporal interventions and comprised of *t*-test’s and Bayesian analyses using the same prior structure as described for the early versus late analyses.

Only if the primary analysis of PCm effects (Δ sham early vs. late TMS) demonstrated reliable effects (*P* < 0.05 or B >3 or <1/3) were further analyses to be implemented in order to investigate alternative explanations. As no such effects were observed, anticipated further analysis was not implemented but the details can be found at https://osf.io/d7uik/.

### Exclusion criteria

Participants were (to be) excluded according to the following criteria:

○ If a participant were to have persistently pressed the same left/right button when they were reflectively unaware of the stimuli, then this strategy would have negated the purpose of the experiment and they would have been excluded from the analyses. This strategy would have been identified by the same left/right discussion being made on 75% or more of the ‘unseen’ trials. No participants were excluded on this basis.
○ If a participant were to have responded negatively to all yes/no questions when under a specific experimental condition (such as active TMS) they would have been excluded, as this would have indicated that their responses were the result of expectation rather than experimental manipulation (Ericsson 2003). No participants were excluded on this basis.
○ If a participant were to give illogical responses (i.e. reporting ‘yes’ to the ‘arrow?’ question and ‘no’ to the ‘something?’ question) on greater than 2.5% of trials, they were to be excluded. One participant was excluded on this basis. As they were only identifiable on the basis of a complete data set, unlike other exclusions, they were included in the 50 reported to have completed the experiment.

If coil repositioning, due to subject’s movement, was required on 50% or more of active TMS blocks, the participants data were to be excluded. 
Other unanticipated technical error may have led to the loss of data and therefore the exclusion of participants. 
Statistical outliers at the participant level were identified and excluded using Chauvenet’s criteria (Taylor 1997). This was applied to each of the critical analyses described above (t-test and Bayesian analysis) for each measure separately. This exclusion therefore only pertained to the measures in question, rather than the removal of the participant's data from all analyses as with other criteria in this section. The application of Chauvenet’s criteria involves describing each analysis as a single vector (as with the Bayesian analysis above). If the likelihood of any data point on that vector, times the number of data points, was <0.5 then that data point was to be excluded from the analysis in question only. Likelihood was defined as the location on a probability distribution function representative of the vector in question. See Supplementary Table S3 for related exclusions. 
Participants may have withdrawn voluntarily from the experiment for any reason.

## Results

Consistent with our baseline hypothesis, TMS applied in isolation at 110 ms after stimulus onset (the BIP) produced evidence of a significant suppression of conscious detection (active vs. sham PrC, *T*_(48)_ = −3.03, *P* = 0.004, mean = −0.08, 95% CI [−0.13, −0.03], *d* = 0.43, BF_uni_ = 12.46, BF_jzs_ = 8.44) while ‘unseen’ discrimination (PcU) remained above chance (with pre-registered exclusions *T*_(40)_ = 6.82, *P* = 3.33 × 10^−8^, mean = 0.65, 95% CI [0.61, 0.69], *d* = 1.07, BF_uni_ = 2.97 × 10^9^, BF_jzs_ = 4.12 × 10^5^, without exclusions *T*_(49)_ = 6.91, *P* = 9.07 × 10^−9^, mean = 0.65, 95% CI [0.61, 0.70], *d* = 0.98, BF_uni_ = 6.51 × 10^9^, BF_jzs_ = 1.38 × 10^6^). However, an exploratory analysis also indicated that the BIP also suppressed PcU compared to sham (*T*_(40)_ = −2.70, *P* = 0.01, mean = −0.09, 95% CI [−0.15, −0.02], *d* = 0.42, BF_jzs_ = 3.97). Although above chance perception in the presence of twice-denied awareness was demonstrated, a complete dissociation of TMS effects between PrC and PcU was not observed, which lessens the extent to which the phenomena might be understood as a form of Blindsight. This may be consistent with previous studies, which have also failed to show a complete dissociation between conscious and residual perceptual capacity (Koivisto *et al.* 2012; Railo *et al.* 2012, 2014; Lloyd *et al.* 2013; Hurme *et al.* 2017, 2019).

**Figure 2.**
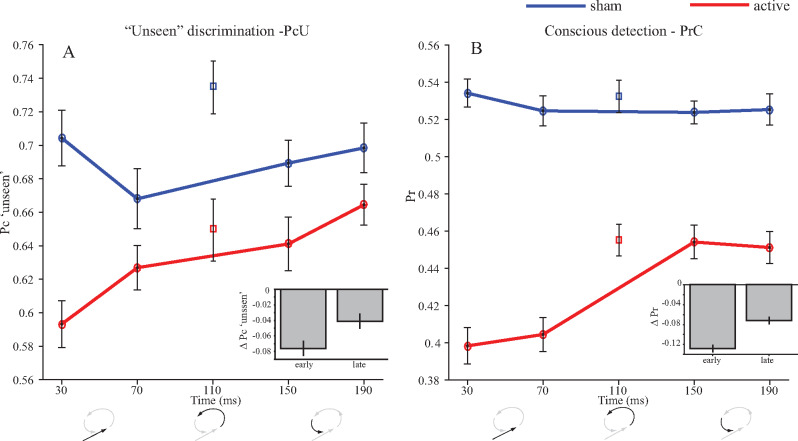
Illustration of group-level results for primary measures. Values on the abscissa denote TMS onset relative to stimuli onset. TMS applied at 110 ms is the BIP applied in isolation. All other pulses were applied in addition to the BIP. Panels **A** and **B**, the primary measures of ‘unseen’ discrimination (PcU) and conscious detection (PrC) and bar inserts collapse data across the early (30 and 70 ms) and late (150 and 190 ms) epochs, illustrating the data to which primary analyses were applied to test temporal order effects. Error bars are the within-subject standard error (Loftus and Masson 1994). Cartoon illustrations at the bottom of the panels indicate the periods most effected by the interventions, corresponding to those depicted in Fig. 1

The primary question of this study was whether ‘unseen’ discrimination was suppressed to a greater extent by early TMS compared with late TMS. This hypothesis was supported, albeit weakly in the initial analyses which collapsed data across the two early and two late interventions (PcU Δ sham early vs. late *T*_(40)_ = −1.93, *P* = 0.06, BF_main_ = 3.82, BF_uni_ = 1.20, BF_jzs_ = 0.90, see Fig. 2A and Table 2). Further pre-registered analyses in which individual TMS onset latencies are analysed separately revealed strong evidence that TMS applied at the earliest time (30 ms) suppressed PcU compared to its later TMS counterpart at 190 ms (*T*_(40)_ = −3.57, *P* = 9.39 × 10^−4^, mean = 0.08, 95% CI [0.34, 0.12], *d* = 0.56, BF_main_ = 89.37, BF_uni_ = 135.95, BF_jzs_ = 32.05, see Fig. 2A). This finding supports the primary hypothesis that residual ‘unseen’ discrimination depends upon the early feed-forward sweep of activity (Lamme *et al.* 2000).

However, contrary to the theory relating conscious awareness to later recurrent processing (Lamme *et al.* 2000), conscious detection was also suppressed to a greater extent by early TMS compared to late TMS (PrC Δ sham early (30 and 70 ms) versus late (150 and 190 ms) *T*_(48)_ = 4.17, *P* = 1.25 × 10^−4^, mean = −0.06, 95% CI [−0.08, −0.03], *d* = 0.60, BF_main(early>late)_ = 0.05, BF_main(late>early)_ = 1.73 × 10^3^, BF_uni(late>early)_ = 408.90, BF_jzs_ = 187.88, see Fig. 2B and Table 2). A direct correspondence between feed-forward/recurrent processing and unconscious/conscious perception was therefore not observed.

**Figure 3.**
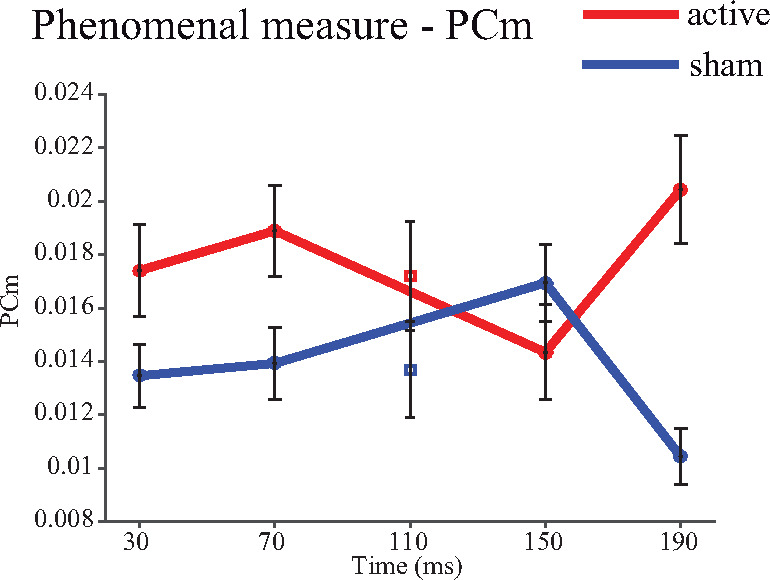
Secondary measure designed to track ‘phenomenal’ consciousness (PCm), in which the numerator consist of trials where awareness is acknowledged (‘yes’ to ‘arrow?’ and ‘something?’ questions) but the responses also indicated a lack of access (a left/right error) and the denominator is all trials where such a response profile was possible. Plot conforms to the same format as Fig. 2

The measure of ‘phenomenal’ consciousness (PCm) was higher under active conditions compared to sham (across times *T*_(47)_ = 2.02, *P* = 0.049, mean = 3.97 × 10^−3^, 95% CI [0.13, 7.91] × 10^−3^, *d* = 0.29 BF_jzs_ = 1.01), but not significantly for the BIP in isolation (*T*_(47)_ = 0.91, *P* = 0.37, mean = 3.50 × 10^−3^, 95% CI [−0.00, 0.01], *d* = 0.13, BF_jzs_ = 0.23). This secondary analysis indicated that participants were more likely to acknowledge awareness of the arrow but report its direction incorrectly during occipital versus sham TMS. Such errors, under Block’s interpretation, can be understood as failures of access consciousness and, therefore, a relative elevation of phenomenal consciousness as indicated by the acknowledgment of awareness. Exploratory analyses indicated that these trends were preserved and their statistical reliability elevated when contributing trial numbers were increased as the alternative measures of ‘phenomenal’ consciousness were applied (see Supplementary Tables S1 and S3 and Methods: Measures). However, the pre-registered analysis revealed no clear evidence that later TMS changed PCm more than early TMS (PCm Δ sham early vs. late *T*_(47)_ = 0.33, *P* = 0.74, BF_main(early>late)_ = 0.91, BF_main(late>early)_ = 0.64, BF_jzs_ = 0.17, see Fig. 3 and Table 2). Therefore, these results did not support the secondary hypothesis that phenomenal consciousness is supported by recurrent processing.

**Figure 4.**
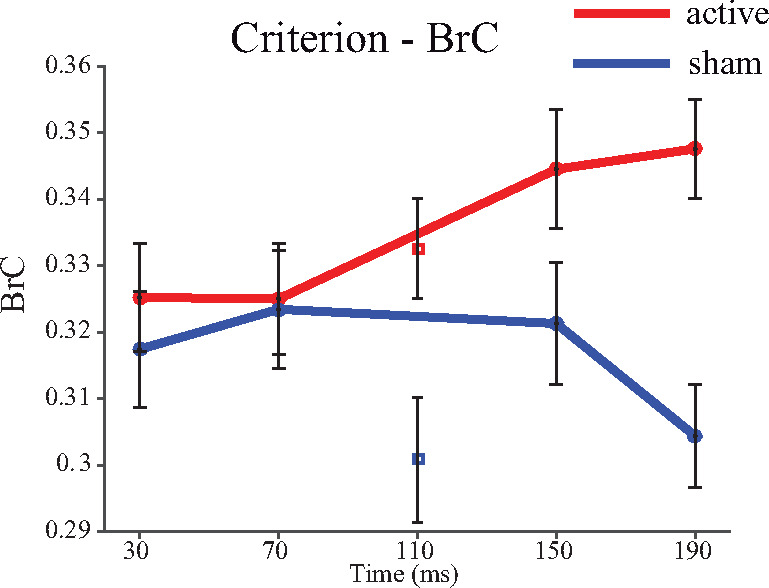
Illustration of exploratory measures of criterion (BrC), which is the non-parametric signal detection theory criterion connate to the PrC sensitivity measure. Plots conform to the same format as Fig. 2

In addition to pre-registered analyses, our design allowed us to derive an exploratory measure of response criterion (BrC), quantifying the participant driven tendency to respond to questions about awareness (Macmillan and Creelman 1990). Recently, response bias has been highlighted as a measure of interest with respect to top-down influences and subjective qualities (Peters *et al.* 2016). Across times there was no clear evidence that participants were any more likely to report awareness irrespective of stimuli under active TMS conditions compared to sham (*T*_(46)_ = 1.41, *P* = 0.17, mean = 0.02, 95% CI [−0.01, 0.05], *d* = 0.20, BF_jzs_ = 0.40). However, in contrast to both primary perceptual measures, which were affected by early TMS, the criterion measure was affected by the application of later TMS compared to early TMS (BrC Δ sham early vs. late *T*_(46)_ = −2.16, *P* = 0.036, BF_main(early>late)_ = 0.34, BF_main(late>early)_ = 3.59, BF_jzs_ = 1.31, see Fig. 4 and Table 2). This indicates that participants were more likely to report awareness independently of what was presented, or had a lower criterion, when active TMS was applied at a later time. Additional exploratory measures are considered in Supplementary Materials.

**Figure 5.**
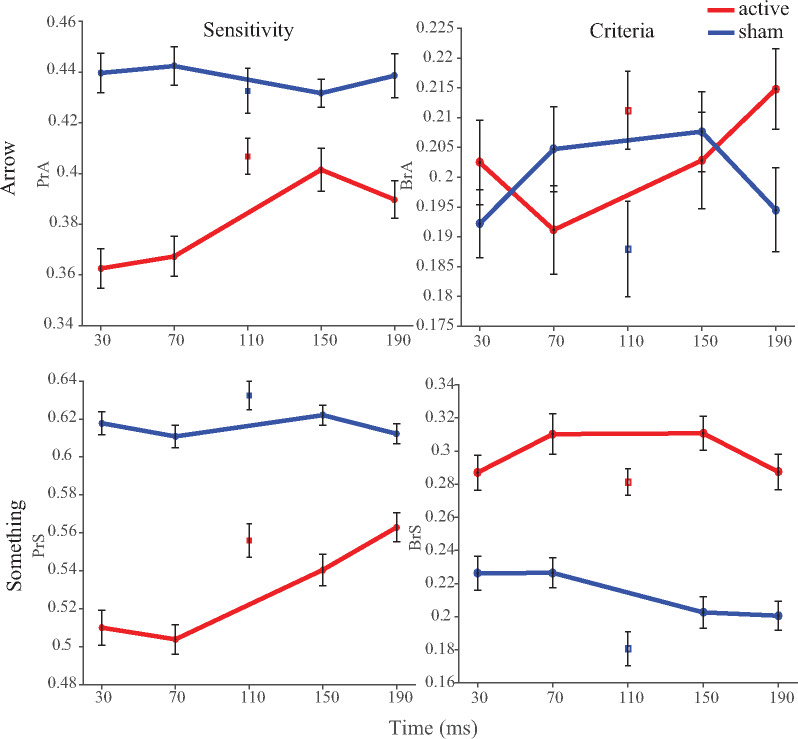
Exploratory analyses of Arrow (A) and Something (S) sensitivity (Pr) and criteria (Br). Plots conform to the same format as Fig. 2

Figure 5 and Table 2 illustrate the exploratory measures of sensitivity and criteria applied to the ‘Arrow?’ and ‘Something?’ questions in isolation. These broadly follow the patterns exhibited in the primary PrC and BrC measures. Potential effects on criteria are slightly reduced in magnitude compared to BrC, likely owing to the reduction in the number of trials contributing to each measure. One noteworthy difference between the BrA and BrS measures is that propensity to respond positively to the ‘Something?’ question is markedly higher than that for the ‘Arrow?’ question under the active TMS condition question, which is to be expected given that the ‘Something?’ question is intended to reflect a lower level of awareness (see participant instructions). It may also indicate that this difference in criteria could be attributed to the lower threshold applied in response to the ‘Something?’ question, and its susceptibility to TMS indicates that it may be a useful adjunct to future applications of related paradigms. Alternatively, this difference between the BrA and BrS measures to TMS, across times, could be the result of participants misinterpreting phosphenes as some form of stimulus, or the TMS introducing some other form of uncertainty.

## Discussion

### Primary hypotheses and measures

Our results support the hypothesis that the early feed-forward sweep of occipital activity supports ‘unseen’ capacity (Lamme and Roelfsema 2000) but are inconsistent with the hypothesis that consciousness is supported by later recurrent processing. Although there is ample evidence that conscious awareness is associated with later recurrent processing (Lamme and Roelfsema 2000; Sergent *et al.* 2005; Pitts *et al.* 2014), there is also evidence linking early components of activity with awareness (Mathewson *et al.* 2009; Railo *et al.* 2011). Here we predicted that later intervention would cause a suppression of the conscious perception measure, but the data indicated that conscious perception also critically depend on early activity. A proponent of the classic theory linking conscious processing to recurrent activity would likely argue that this is to be expected, as the later recurrent phase is dependent upon the initial forward sweep of information. The feed-forward sweep could therefore be understood as enabling or preparatory (Marr 1982; Allen *et al.* 2014) rather than constitutive of conscious awareness, and so disruption of the feed-forward input could be expected to interfere with later conscious processing, particularly as TMS effects only carry forward in time. In this way, the later phase might still constitute the conscious process. However, such a distinction between merely enabling conscious processing and constituting it is difficult to maintain. Our data suggest that conscious detection depends upon early processing to a greater extent than on later processing, suggesting that early feed-forward activity may play a more important causal role. If this is so, it is difficult to see why early activity should not be as tightly associated with conscious perception as later recurrent activity.

Residual ‘unseen’ capacity has appeared to be resilient to TMS interventions (Boyer *et al.* 2005; Allen 2012; Allen *et al.* 2014; Koenig and Ro 2019), although one study has reported perturbation with TMS at 60 ms post-stimulus (Hurme *et al.* 2017). The resilience of residual capacity, together with the evocation of TMS-induced blindsight, motivated the application of the BIP on all trials, which, in combination with relatively high trial numbers and sample sizes, allowed us to map the temporal dependencies of ‘unseen’ sensitivity. However, TMS pulses carry their effects forward in time and the presence of the BIP meant that, to some extent, recurrent activity was interfered with under all active conditions. The difference between early and late conditions is therefore a relative one. Recurrent processing is affected in all conditions, but the effects on conscious detection and ‘unseen’ sensitivity were affected to a greater extent when early feed-forward processing was disrupted compared to TMS applied later on.

Both conscious detection and ‘unseen’ discrimination sensitivity appeared to be most susceptible to early interventions, and both were susceptible to the BIP in isolation. Whilst the susceptibility of ‘unseen’ discrimination to interventions can in part be attributed to relatively well numerated data, it also possibly suggests that they are supported by a common perceptual mechanism and differences between these two measures could be interpreted as differences in subjective thresholds and approaches to the task (Peters and Lau 2015).

### Criteria measures of consciousness

The effects of TMS upon criterion/bias dissociated temporally from the primary measures, with later TMS, as opposed to early TMS, being the more effective intervention. How then is bias, and the kind of false alarm error which drives it, to be understood? Bias, operationalized as the propensity to report awareness, might reflect a form of awareness that is independent of perception, and the contrast between bias (also understood as subjective criterion) and objective perception could be fruitful in uncovering the top-down, dispositional and subjective aspects of consciousness (Peters *et al.* 2016). If the criterion measure tracks some form of subjective awareness or interpretation, the later TMS effects could support Lamme’s framework. Indeed, under the ‘neural stance’ (Lamme 2006a) the measure of criterion should be understood as relating to consciousness to a greater extent than the detection measures precisely because it is affected by later TMS, although such a position could be criticized for circularity (Kouider *et al.* 2010).

Until now, relatively few examples of dissociations involving criterion have received attention, and investigations have more commonly focused on the problems of bias and the need to remove criterion from measures (Snodgrass *et al.* 2004; Lloyd *et al.* 2013; Balsdon and Azzopardi 2015). Although there are circumstances where removing subjective criteria is useful (e.g. when comparing perception across individuals or looking at changes in strategy over time), when the focus of the enquiry relates to consciousness, removing the subjective element could be counterproductive (Lau 2008). Although exploratory, the current observations of a causal, temporal dissociation where perceptual measures are affected early and bias was affected later (cf. Fig. 2 vs. Fig. 4) has the potential to inform our understanding of conscious processes. Later TMS increased reported awareness independently of perception. Whether this lowering of criterion by later TMS is the result of suppression of the threshold for entry into consciousness (i.e. disruption of suppression) or corresponds to mistakes in interpreting noisy percepts, is unclear based on the current data and may be an area for future replication and investigation. However, it does seem that the later effects relate to a high-order effect upon the conscious report, rather than the perceptual or access system, involving a disposition to report awareness independently of the stimuli.

### Phenomenal measures

With respect to the ‘phenomenal’ measures, the hypothesis that an additional TMS pulse applied during later processing should disrupt ‘phenomenal’ consciousness to a greater extent than earlier TMS was not supported. The measures of phenomenal consciousness did not show a temporal dissociation. However, as described in our pre-registered protocol (https://osf.io/d7uik/), TMS applied at the BIP and therefore under all active TMS conditions, may have disrupted recurrent processing (Lamme 2006b). If so the current findings that participants are *more* likely to exhibit responses consistent with relative phenomenal consciousness without access consciousness, in the context of disrupted recurrent activity, may be difficult to reconcile with Block’s (2005, 2007) theory.

However, we acknowledge that our characterization of ‘phenomenal’ measures may not reflect the kinds of responses Block intends in his descriptions of phenomenal consciousness. Indeed, it is possible that the apparent richness of phenomenal content corresponds to the detail and granularity of the experimental questions posed and that our questioning procedure failed to capture Block’s intention. An alternative explanation of the data is that under active TMS conditions, aspects of the cognitive processing associated with access consciousness (decision-making, report) are affected such that (as above) participants are in general more likely to report awareness and make an error in, e.g. reporting direction, which inflates the PcM measures. That is, the increase in ‘phenomenal’ measures during disrupted recurrent processing does not mean that there was a higher proportion of cases of phenomenal consciousness without access consciousness, but instead that there was a higher proportion of cases of ‘abnormal’ access reports. In this way, we observe changes in criterion or access that happen to fit the PcM profile. This would not be inconsistent with Block’s claim that recurrent processing is essential for phenomenal consciousness.

### Predictive coding interpretation

The observed late effect of TMS on criterion can also be interpreted under, and potentially inform, theories of neural predictive coding. In brief, these theories hold that there are prior sets of predictions, which are then integrated with incoming information according to Bayesian principles, resulting in prediction error or free-energy which the system attempts to minimize. The results of this integration form a posterior which can then become the prior for the subsequent series of events (Friston 2010, 2012; Clark 2013). Predictive coding theories are notoriously difficult to falsify owing to their alignment with alternative, well-established theories (e.g. attention or learning, Bowers and Davis 2012). One of the few areas in which there appears to be unique and therefore testable predictions is in the temporal order of events – the prior comes first. The prior, being the state of the system independent of the incoming information, might be seen as most closely resembling the measures of criterion, as it also reflects the tendency to respond independently of incoming information. As criterion was affected at a later time, but the predictive coding framework would suggest its earlier involvement, there is a discrepancy that could be seen as contrary to the framework. Ameliorating explanations are possible. For example, later TMS could affect the integration process resulting in posteriors that are more dependent upon the prior, as opposed to the incoming information, leading to increased false alarms. However, such an effect should have also led to a comparable loss of sensitivity under late TMS, which was not observed, although it is possible that it could have been obscured by the dominance of the early effect. Similarly, prior expectation and report contents might dissociate in other unanticipated ways or the relevant priors may be independent of activity effected by the TMS. Even though these possibilities are speculative interpretations of exploratory results we feel they are informative and worthy of note as they offer avenues for future enquiry and the discrepancy between the simplest interpretation – of top-down criterion relating to the prior – may be contrary to predictive coding, where unique testable predictions are sparse.

### Signal detection theory considerations

The range of methods applied to investigate the presence of unconscious processing and related blindsight-type phenomena is a matter of keen debate (Schmidt 2007; Peters *et al.* 2017). Here the decision was made to place the distinction between presence and absence of awareness in the hands of the participant where the PcU measures drew on responses where participants reported and confirmed an absence of awareness (Cheesman and Merikle 1987; Varela 1996; Weiskrantz 1997; Dienes 2004). Measures relating to the presence of consciousness (PrC and BrC) here, and more widely within the field, centre upon SDT and to some extent the dissociation between sensitivity and criterion justifies the use of SDT. However, we wish to highlight a difficulty with the application of SDT within the context of consciousness research and particularly with respect to confidence or second-order judgements. A central assumption in the application of both classical and second-order SDT is that underlying processes are best described by a pair of distributions where a noise distribution is of comparable magnitude to that of a signal distribution. Yet participants rarely claim awareness of stimuli that were not presented. From an evolutionary perspective, metacognitive systems that minimize drawing from such an error distribution might be successful. This might suggest that the noise distribution should not be assumed to be comparable to that of the signal, especially with respect to second-order judgements. These issues are exacerbated by the parametric normalization applied to the most commonly applied measures in this field (Green and Swets 1966). Such measures are subject to gross distortion by low false alarm rates because normalized metrics approach infinity as false alarm rates tend towards zero (here 111 cells out of 500 contained five or fewer false alarms where there where 96 opportunities for false alarms per cell). The work-around often recommended (Macmillan *et al.* 1990; Hautus 1995) is the addition of a constant to both hit and false alarm rates, or estimating false alarms rates for participants who did not make false alarms from those that did (Lee 2008; Fleming 2017). Although estimating reports based on other participants is preferable, within the context of consciousness research and epistemically, this work-around is highly problematic. It essentially involves the analyst generating pseudo-responses that participants did not make in order to apply their paradigm of choice. Avoidance of this issue and dependency on the assumption of any particular distribution were motivating factors for the *a priori* adoption of non-parametric SDT (Corwin 1994). Although some model comparison techniques are limited by the use of non-parametric SDT, the similarity between the primary non-parametric measures applied here (PrC and BrC, Fig. 2) and the classical parametric measures (dʹ and c, See Supplementary Fig. S3) suggests the utility of the parametric assumption may be minimal. We therefore recommend the broader adoption of non-parametric SDT in consciousness research.

### Blindsight

This paradigm was designed to be capable of demonstrating blindsight-type phenomena, where blindsight is interpreted as a dissociation (Weiskrantz 1992) between conscious awareness of stimuli and residual (unconscious) perceptual capacity. A potential limitation for this paradigm may be that the residual, reportedly ‘unseen’, capacity cannot be attributed to the TMS intervention, as above chance performance is present at baseline, and therefore is less than perfectly analogous to lesion-based blindsight where the residual capacity is only apparent with the lesion. Furthermore, it is possible that the existence of blindsight and unconscious perception more generally, may be brought into question through the application of a more nuanced, less binary, questioning procedure. The distinction between unconscious and pre-reflective capacity (Petitmengin and Bitbol 2009) and the role of bias in report are important areas for development in future research. The current study, however, did not demonstrate a blindsight-type dissociation. Had the original hypothesis of a temporal dissociation been observed, where PrC would have been affected at later times in comparisons to PcU, then such a pattern may have resembled blindsight. Instead, the temporal dissociation observed between the perceptual and the criteria measures tentatively suggests that feed-forward and recurrent phases more closely reflect perception and report, as opposed to the unconscious and conscious (blindsight) dichotomy.

## Conclusions

This investigation sought to investigate the role of feed-forward and recurrent processing in relation to visual consciousness. We found that both measures of reportedly ‘unseen’ perception and conscious detection were affected to the greatest extent by the application of early TMS, indicating that they both critically depend upon early feed-forward processing. Exploratory analysis indicated that these perceptual measures dissociated from criteria-related measures that appeared to be affected by later TMS. These findings suggest that rather than the feed-forward recurrent distinction mapping onto to the difference between conscious and unconscious processing, the most parsimonious interpretation of the current findings is that early visual processing is more perceptual, whereas later recurrent processing relates to top-down, subjective or dispositional aspects of responding.

## Supplementary data

Supplementary data are available at *NCONSC Journal* online and at the online data repository https://osf.io/dwfqv/.

## Supplementary Material

niaa015_Supplementary_Data
