# Supplementary material for: Causal manipulation of feed-forward and recurrent processing differentially affects measures of consciousness
Source: Neurosci Conscious. 2020 Sep 7;2020(1):niaa015. doi: 10.1093/nc/niaa015 (PMC7475771; doi:10.1093/nc/niaa015)
Supplement: niaa015_Supplementary_Data [file niaa015_supplementary_data.zip › NC_Supplementary_16July20.docx]

Supplementary Material for: Causal manipulation of feed-forward and recurrent processing differentially affects measures of consciousness

Christopher Allen, Tommaso Viola, Elizabeth Irvine, Jemma Sedgmond, Heidi Castle, Richard Gray, Christopher D. Chambers

**Supplementary Methods**

The phenomenal consciousness measure (PCm)

At pre-registration we anticipated practical considerations, which may have constrained the allocation of response conditions to the PCm measures. These led to three sets of measures (see Table S1) which contained incrementally more trials conditions and were to be sequentially applied, in the event of one measure containing insufficient data to probe relationships between Block's phenomenal consciousness upon recurrent processing. As it transpired it was possible to use the first (PCmA) measure as presented in the main text, as PCm.

There were two critical attributes that the PCm should express: i) subjects acknowledge awareness, which is an expression that there is 'something it is like' - phenomenal consciousness, and ii) with respect to the same experience, participants, access to information is limited, evidenced by incorrect responses to arrow direction or stimuli presence. All the measures described in table S1 conform to these requirements, therefore if Block's prediction were correct, we should expect lower levels of these measures when TMS was applied late compared to when applied early, as recurrent activity and phenomenal consciousness would be disrupted.

|  | Numerator | | | | Denominator | Order of |
| --- | --- | --- | --- | --- | --- | --- |
| Measure | Stimulus | Something? | Arrow? | L/R Discrimination |  | preference |
| PCmA | Arrow | Yes | Yes | Incorrect | Arrow present | 1 |
| PCmB | Arrow | Yes | Yes | Incorrect | Arrow present | 2 |
|  |  | Yes | No |  |  |  |
| PCmC | Arrow | Yes | Yes | Incorrect | all trials | 3 |
|  |  | Yes | No |  |  |  |
|  | Non-Arrow | Yes | Yes | N/A |  |  |
|  | Nothing | Yes | Yes |  |  |  |
|  |  | Yes | No |  |  |  |

**Table S1**. The three possible response category allocations used to derive the phenomenal consciousness measure (PCm). The final selection of the PCmA measure as representative of ‘phenomenal’ consciousness was determined according to the numbers of contributing trials.

Before inferential statistics were applied to the PCm measure, the measure itself had to be selected as one of the three response category allocations described in Table S1. The criteria for selection depended on the incidence of trials contributing to the numerator of the measure. There were four important data points per participant (active early, active late, sham early, sham late). It was possible that Block's prediction could have been fulfilled when only two of these four cells contain non-zero data, i.e. if the phenomenal behavioural pattern was expressed only under sham TMS conditions, when recurrent processing was not disrupted by the BIP. Therefore, participant’s data was excluded when three or four of these cells are empty. This was in addition to the exclusion of individual participants data according to Chauvenet's criteria etc. (see Exclusion Criteria). Following such exclusions, if the number of contributing subjects fell below half of its original value, the next measure of preference in Table S1 should have been used. This left open the possibility that up to half the data points of the main group level analysis could have contained zero values. Since it has been previously demonstrated that t-tests can tolerate similar levels of skewness (Sullivan & D’Agostino, 1992) and Block’s hypothesis may have been informed by such a pattern, we were justified in the application of such tests. The exclusion of individual participants’ data in terms of PCm only effected the PCm analysis so it was not relevant to the exclusion criteria set out in the main text. In the event, anticipating these contingencies was unnecessary as the first measure of Table S1 (PCmA) fulfilled the criteria and was used as the main PCm measure. For completeness and transparency, analyses applied to the other PCm measures have been described in Supplementary Results.

Arrow and Something measure (PrA, BrA, PrS, PrS)

Table S2 describes the signal detection theory class allocation for the measures of sensitivity and bias specifically for the presence of the arrow stimuli, assayed via the ‘Arrow?’ question, and, separately, the presence of the arrow or non-arrow as assayed by the ‘Something?’ question.

| Measure | Stimulus | Response | SDT Class |
| --- | --- | --- | --- |
| PrA, BrA | Arrow | Yes | HIT |
|  |  | No | MISS |
|  | Non-arrow | Yes | FA |
|  |  | No | CR |
|  | Nothing | Yes | FA |
|  |  | No | CR |
| PrS, BrS | Arrow | Yes | HIT |
|  |  | No | MISS |
|  | Non-arrow | Yes | HIT |
|  |  | No | MISS |
|  | Nothing | Yes | FA |
|  |  | No | CR |

**Table S2**. Signal detection theory (SDT) classes for measures of Arrow sensitivity (PrA), and bias (BrA) and Something sensitivity (PrS), and bias (BrS). FA = False Alarms; CR = Correct Rejections.

**Supplementary Results**

In addition to the analyses described in the main text, our design allowed us to derive sets of exploratory measures that are potentially informative about different aspects of conscious report in relation to the feed-forward and recurrent interventions and align the current investigation with commonly used measures in the field. Temporal comparisons applied to these measures are summarised in table S3 and follow the pre-registered analyses applied to the non-measure specific analyses. In addition to these, we present analyses applied to the alternative pre-registered measures of phenomenal consciousness (see above).

|  | Measure | T | p | df | mean | 95% CI | | *d* | BFmain | BFmain | BFuni | BFuni | BFjzs | Number |
| --- | --- | --- | --- | --- | --- | --- | --- | --- | --- | --- | --- | --- | --- | --- |
|  |  |  |  |  |  |  |  |  | early>late | late>early | early>late | late>early |  | outliers |
| primary | PcU | -1.93 | 0.06 | 40 | -0.03 | -0.07 | 0.00 | -0.30 | **3.82** | 0.19 | 1.20 | 0.03 | 0.90 | 1 |
|  | PrC | -4.17 | 0.00 | 48 | -0.06 | -0.08 | -0.03 | -0.60 | 1734.41 | **0.05** | 408.90 | 0.01 | 187.88 | 0 |
| secondary | PCmA | 0.33 | 0.74 | 47 | 0.00 | 0.00 | 0.01 | 0.05 | 0.64 | 0.91 | NA | NA | 0.17 | 1 |
|  | PCmB | 0.03 | 0.97 | 46 | 0.00 | -0.01 | 0.01 | 0.01 | 0.55 | 0.58 | NA | NA | 0.16 | 2 |
|  | PCmC | 0.17 | 0.87 | 46 | 0.00 | -0.01 | 0.01 | 0.02 | 0.35 | 0.45 | NA | NA | 0.16 | 2 |
| exploratory | BrC | -2.16 | 0.04 | 46 | -0.03 | -0.06 | 0.00 | -0.32 | 3.59 | 0.34 | NA | NA | 1.31 | 2 |
|  | type 1 | -1.01 | 0.32 | 48 | -0.06 | -0.19 | 0.06 | -0.14 | 1.32 | 0.66 | NA | NA | 0.25 | 0 |
|  | type 2 | -0.94 | 0.35 | 47 | -0.02 | -0.06 | 0.02 | -0.14 | 1.31 | 0.44 | NA | NA | 0.24 | 1 |
|  | meta diff | 0.73 | 0.47 | 47 | 0.04 | -0.06 | 0.14 | 0.11 | 0.93 | 1.06 | NA | NA | 0.20 | 1 |
|  | d' | -2.64 | 0.01 | 45 | -0.13 | -0.23 | -0.03 | -0.39 | 14.33 | 0.08 | NA | NA | 3.51 | 0 |
|  | c | 2.60 | 0.01 | 45 | 0.09 | 0.02 | 0.16 | 0.38 | 0.59 | 1.90 | NA | NA | 3.22 | 0 |
|  | d' Bayes | -3.02 | 0.00 | 47 | -0.14 | -0.23 | -0.05 | -0.44 | 37.87 | 0.08 | NA | NA | 0.23 | 1 |
|  | c Bayes | 2.79 | 0.01 | 47 | 0.09 | 0.02 | 0.15 | 0.40 | 0.62 | 1.78 | NA | NA | 4.84 | 1 |
|  | HR | -3.81 | 0.00 | 46 | -0.06 | -0.08 | -0.03 | -0.56 | 121.60 | 0.14 | NA | NA | 64.38 | 2 |
|  | FAR | 0.47 | 0.64 | 47 | 0.00 | -0.01 | 0.02 | 0.07 | 0.31 | 0.63 | NA | NA | 0.17 | 1 |
|  | LR YN | -0.02 | 0.99 | 48 | 0.00 | -0.06 | 0.06 | 0.00 | 0.78 | 0.77 | NA | NA | 0.16 | 0 |
|  | LR YY | -1.93 | 0.06 | 46 | -0.01 | -0.02 | 0.00 | -0.28 | 3.41 | 0.29 | NA | NA | 0.87 | 2 |
|  | PrA | -2.96 | 0.00 | 46 | -0.04 | -0.06 | -0.06 | -0.43 | 29.96 | 0.11 | NA | NA | 7.12 | 2 |
|  | BrA | -0.73 | 0.47 | 46 | -0.01 | -0.04 | -0.04 | -0.11 | 1.14 | 0.83 | NA | NA | 0.20 | 2 |
|  | PrS | -4.12 | 0.00 | 47 | -0.04 | -0.06 | -0.06 | -0.59 | 1415.17 | 0.05 | NA | NA | 156.92 | 1 |
|  | BrS | -1.42 | 0.16 | 47 | -0.03 | -0.06 | -0.06 | -0.20 | 1.61 | 0.17 | NA | NA | 0.40 | 1 |

**Table S3**. Summary of temporal order comparisons applied to *all* measures, including exploratory and secondary analyses. This table conforms to the same format as Table 2 of the main text. Additionally, type 1, type 2 and meta diff refer to meta-d’ related measures (see Meta-d’ section below and Fleming, 2017; Fleming & Lau, 2014), d’ and c refer to classic parametric signal detection measures (see d’ and c below and Green & Swets, 1966), PCmA-C refer to the alternative measures of phenomenal consciousness (see Phenomenal Consciousness measures below). d’ and c refer to the application of classic SDT metrics and are accompanied by comparable measures derived through the hierarchical Bayesian (Bayes) approach set out by Lee (2008). HR is Hit Rate and FAR is False Alarm Rate. LR YN refers to Left Right discrimination when participant report being aware of something but not the arrow and LR YY refers to discrimination when participants report full awareness. PrA, PrS, BrA and BrS refer to measures of sensitivity (Pr) and criteria (Br) to the Arrow (A) and Something (S) stimuli.

**Phenomenal Consciousness measures A-C**

For completeness, the alternative measures of phenomenal consciousness (described in above) are depicted in Figure S1 and temporal comparison statistics are described in Table S3. From measure A to C the contributing conditions, which conform to ‘phenomenal’ consciousness, expand and so the numbers of contributing trials increase. No evidence of a temporally specific dissociation was observed.

**
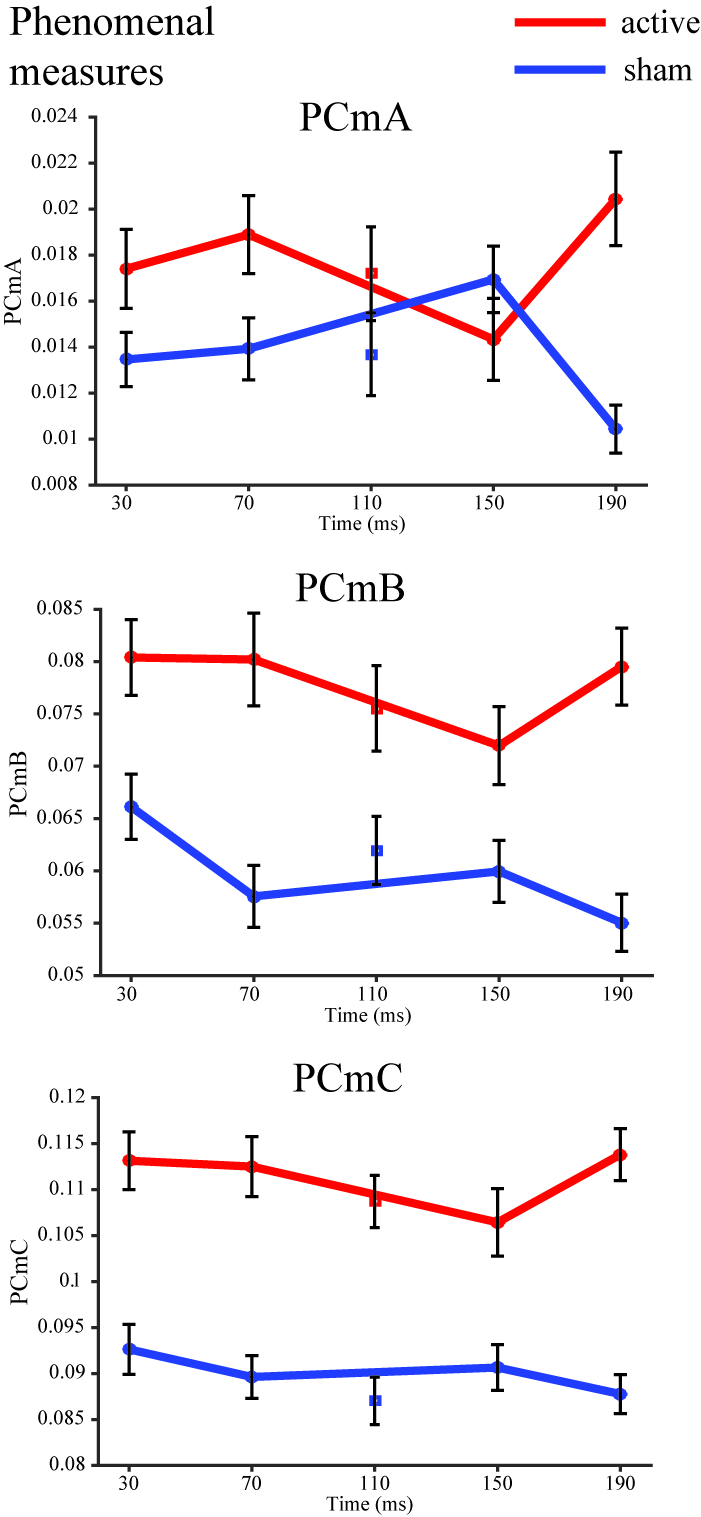
**

**Figure S1.** Full exposition of secondary measures designed to track ‘phenomenal’ consciousness, in which the numerators consist of trials where awareness is acknowledged but the responses also indicated a lack of access (e.g. for A, a left/right error) and the denominators are all trials where such a response profile was possible. Measures A to C become more liberal through the inclusion of more trials conforming to this general pattern (see above and Table S1). The primary measure for statistical purposes is PCmA where the first panel of this figure reproduces figure 3 of the main text (also see Table S2). Plots conform to the same format as figure 2.

**Meta-d’**

The first supplementary exploratory measure of interest is meta-d’, which is described as reflecting metacognitive sensitivity, here applied to the questions which probe reported conscious awareness of the stimuli (type 2 or meta sensitivity), and which considers the difference between this level of responding and type 1 or left / right perceptual sensitivity. As these measures have recently become widely used within dissociation paradigms similar to this, it allows for a more direct interpretation in relation to recent literature (Fleming, 2017; Fleming & Lau, 2014). Appling this measure to the current task involved treating the detection questions as confidence ratings, and the directional response was the forced choice discrimination. The highest level of confidence was when participants responded ‘Yes’ to both the ‘arrow?’ and the ‘something?’ question. The next level was responding positively to the ‘something?’ question only and the lowest level was responding ‘no’ to both questions. It is however worth highlighting that this task was not designed for such application and there were often instances where participants made no directional mistakes when reporting a higher levels of awareness. This made the application of previous iterations of the meta-d’ approach problematic for reasons described in the discussion and it was only with the publication of the most recent hierarchical Bayesian approach to its application (Fleming, 2017) that the results became interpretable, through the use of group level estimations of responses.

Broadly, the results showed trends in line with the observations previously reported but with much lower effect sizes; Type-2 meta-d’ revealed a reliable difference between active and sham conditions (T_(47)_=3.01, p=0.004, BF_jzs_=8.14) in which participants were more likely to report awareness under the active condition, corresponding to the criterion (BrC) finding. All other comparisons involving related measures and including temporal comparisons did not clearly support specific effects (T_(47)_<1.01, p>0.32, BF<1.06, see Figure S2 and Table S3).


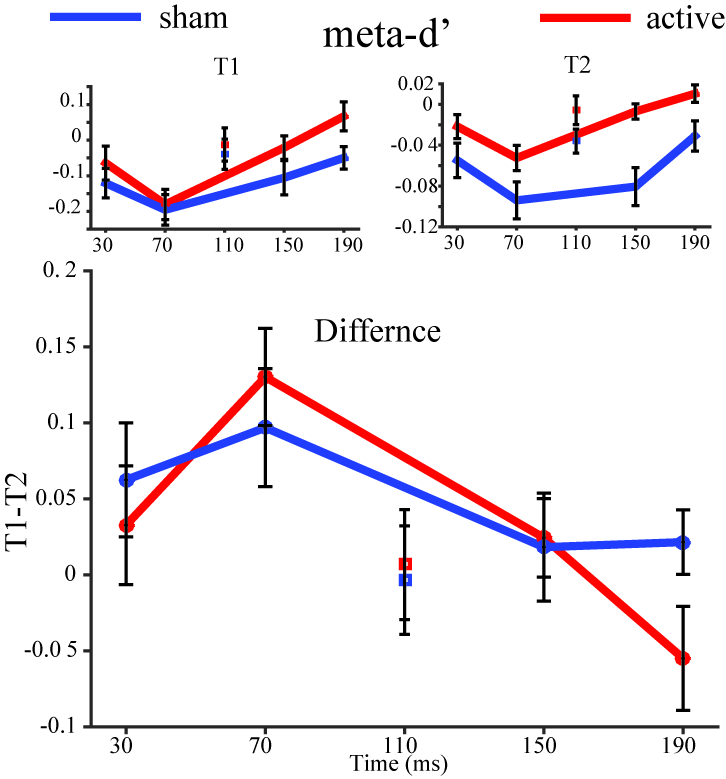


**Figure S2.** Meta-d’ related measures where T1 refers to type 1 perceptual sensitivity and T2 refers to type 2 or metacognitive sensitivity and T1-T2 illustrates the difference between the two. Plots conform to the same format as figure 2.

**d’ and c**

To provide a more direct comparison with the wider psychophysical literature, classic signal detection theory measures of d’ (sensitivity) and c (criterion) were calculated using the same signal detection categorisations as PrC and BrC (Corwin, 1994; Green & Swets, 1966; Macmillan et al., 1990). Here where false alarm rates are zero in any cell, the participant was excluded for the corresponding classic analyses (six participants, also see discussion).

These exploratory analyses revealed effects in line with the preregistered analyses. Active TMS suppressed d’ relative to sham (T_(45)_=3.75, p=5.07×10^-4^, BF_jzs_=53.79, see Figure S3) across times, without influencing c (T_(45)_=0.57, p= 0.57, BF_jzs_=0.19), likely due to a crossover interaction with time (see Figure S3). As with non-parametric measures, TMS produced time-dependent effects on both d’ and c, with d’- reduced to a greater extent by early TMS compared with late TMS (T_(45)_=-2.64, p=0.01, BF_jzs_=3.51) and c-criterion lower during late TMS compared with early TMS (T_(45)_=2.60, p=0.01, BF_jzs_=3.22, see Table S3).


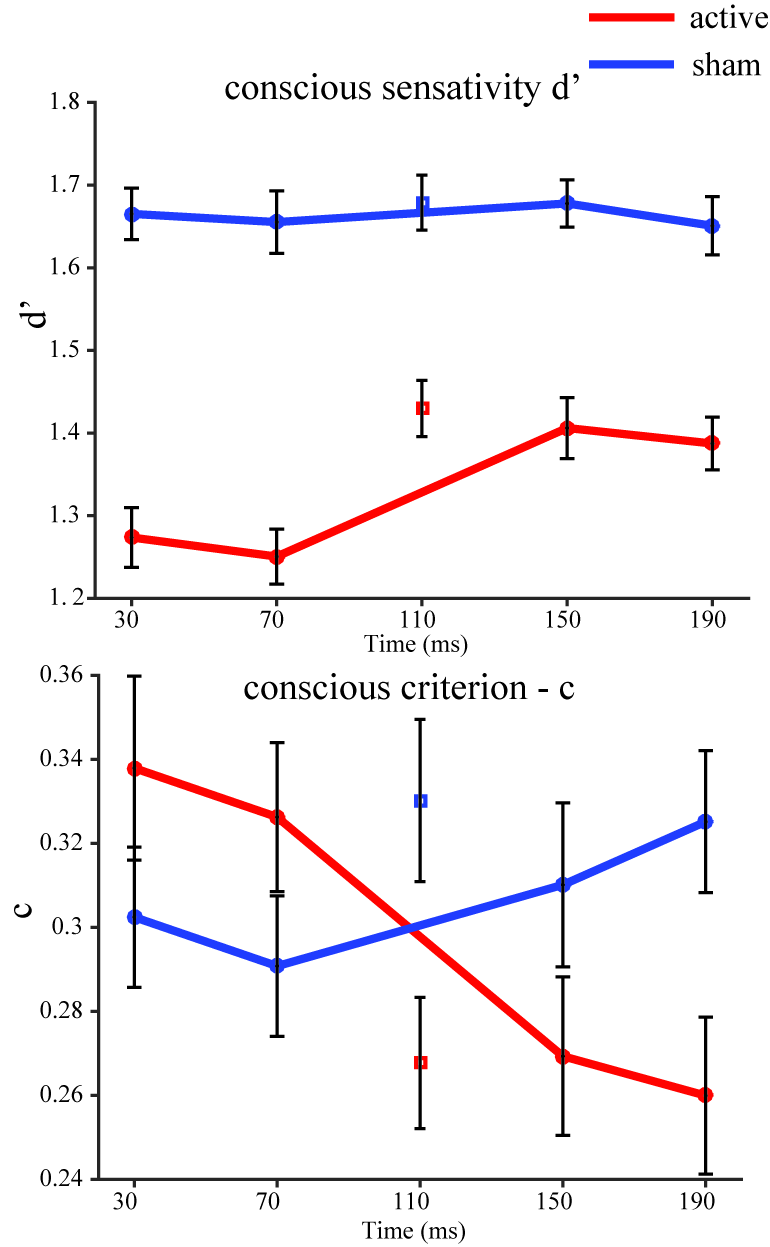


**Figure S3.** Classic parametric signal detection theory equivalent of conscious sensitivity PrC – d’ and conscious criterion BrC – c. Plots conform to the same format as figure 2.

**Hierarchical Bayes d’ and c**

It is possible to apply a hierarchical Bayesian approach to produce classic signal detection measures d’ and c (Lee, 2008; Lee & Wagenmakers, 2014). This has the advantage of not being as susceptible to empty cell issues in comparison to classic measures (see discussion), as estimates are arrived at using group means. Additionally, this method aligns the classic measures with the method applied to the Meta-d’ measures above. The current implementation follows the methods described in (Lee, 2008) where bin size and sample repletion level was set to 1000.

The results closely follow the classic measure described above (c.f. figures S3 and S4). The hierarchical Bayesian version of d’ showed a difference between active and sham conditions across times (T_(47)_=-3.78, p=4.45×10^-4^, mean=-0.27, 95% CI [-0.42,-0.13], *d*=-0.55, BF_jzs_=59.73). The change from sham data showed significant early suppression times (Bayes SDT d’ Δ sham early vs. late T_(47)_=-3.02, p=0.00, BF_jzs_=8.23, see table S3). The measure of bias c also showed a difference between active and sham conditions across times (T_(47)_=-2.08, p=0.04, mean=0.07, 95% CI [-0.14,0.00], *d*=-0.31, BF_jzs_=1.13) and a differential effect between early and later times (Bayes SDT c Δ sham early vs. late T_(47)_=2.79, p=0.01, BF_jzs_=4.84, see table S3)

**
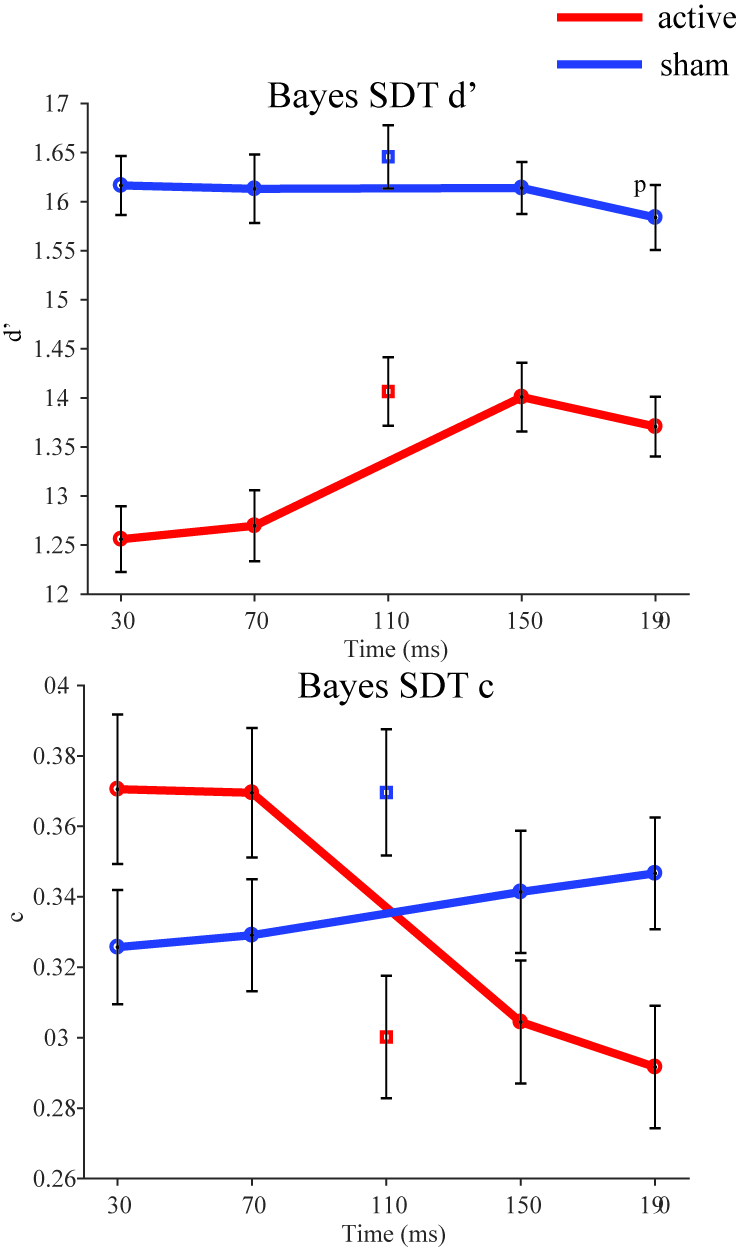
**

**Figure S4.** Parametric signal detection theory equivalent of conscious sensitivity PrC – d’ and conscious criterion BrC – c derived using hierarchical Bayesian approach (Lee, 2008). Plots conform to the same format as figure 2.

**HR and FAR**

In addition, to the measures already discussed, we applied the same analyses to the constituents of the conscious detection measures, i.e. hit rates (HR) and false alarm rates (FAR). Figure S5 illustrates the outcomes of these analyses, where FAR showed a significant elevation under active TMS relative to shame across times (T_(47)_=5.01, p=8.12×10^-6^, mean=0.03, 95% CI [0.02,0.05], *d*=0.72, BF_jzs_=2344) and HR showed a weaker but significant reduction (T_(46)_=-2.08, p=0.04, mean=-0.04, 95% CI [-0.08,0.00], *d*=-0.30, BF_jzs_=1.13). However, FAR did not appear to show a clear time dependency (FAR Δ sham early vs. late T_(47)_=0.47, p=0.64, BF_jzs_=0.17, see table S3), whereas HR did (HR Δ sham early vs. late T_(46)_=-3.81, p=4.12×10^-4^, BF_jzs_=64.38, see table S3) which clearly drives the early effects on conscious detection.


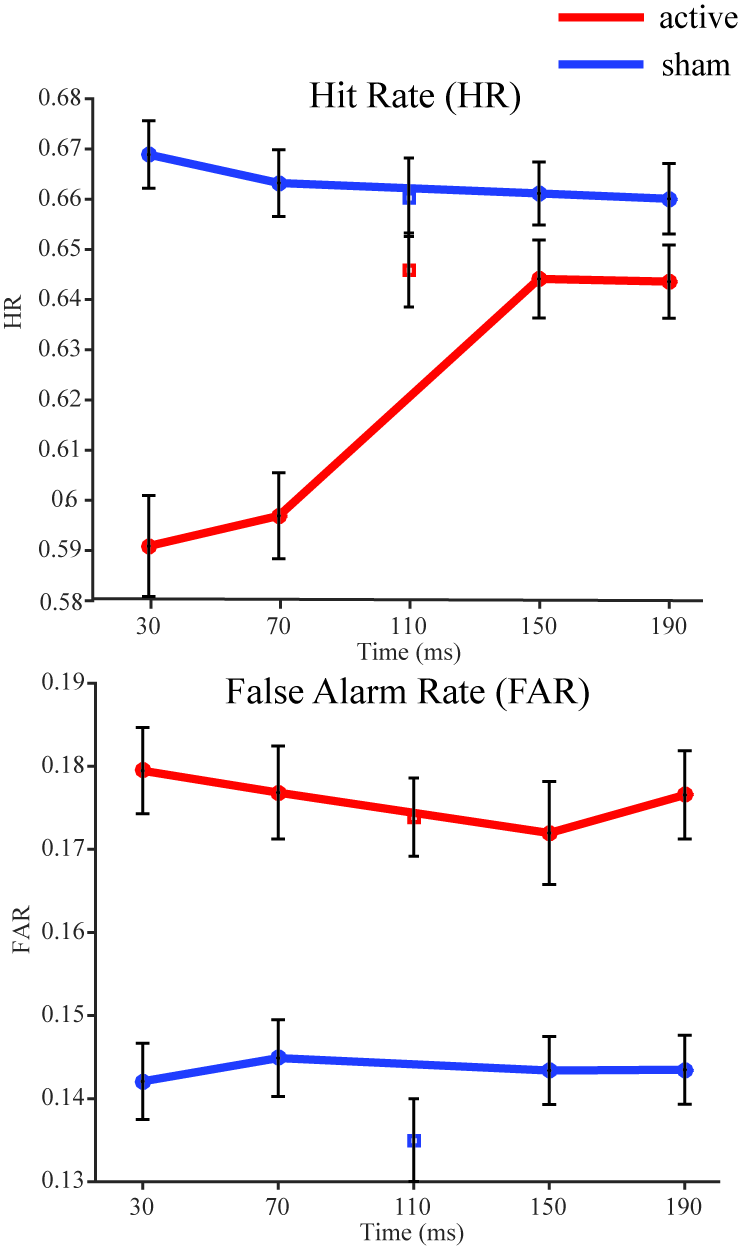


**Figure S5.** Hit Rates (A) and False Alarm Rates (B) for the conscious detection measures, where Hit Rate = Hits/(Hits + Misses) and False Alarm Rates = False Alarms / (False Alarms + Correct Rejections), see table 2 for further specification. Plots conform to the same format as figure 2.

**Discrimination under all levels of detection**

This section describes left/right discrimination at the three levels of reported awareness and applies the standard analyses applied to other measures.

When participants were fully aware of the stimuli discrimination capacity was reduced under active TMS compared to sham across times (T_(46)_=-2.76, p=0.01, mean=-0.01, 95% CI [-0.02.-0.00], *d*=-0.40, BF_jzs_=4.54) and may have been effected more by the relatively early intervention (Pc “Yes” something “Yes” arrow Δ sham early vs. late T_(48)_=-1.93 p=0.06, BF_jzs_=0.87, see table S3), potentially reflecting the PrC detection measure, although note that this measure was affected by celling effects (see figure S6).

When participant acknowledged partial awareness of the stimuli (“Yes” to something and “No” to arrow) there appeared to be relatively small differences between active and sham conditions where participants got direction incorrect more often under the active condition compared to sham across times (T_(47)_=-2.39, p=0.02, mean=-0.05, 95% CI [-0.09.-0.01], *d*=-0.34, BF_jzs_=2.02), but there did not appear to be a temporal dependency of any such difference (Pc “Yes” something “No” arrow Δ sham early vs. late T_(48)_=-0.02, p=0.99, BF_jzs_=0.16, see table S3). This measure makes up the numerator of the PCm measure, so closely resembles it in terms of data patterning (c.f. Figures S6 and figure 3).

The results of applying analyses where participants respond “No” to both awareness probes are discussed in the main text as the PcU measure.


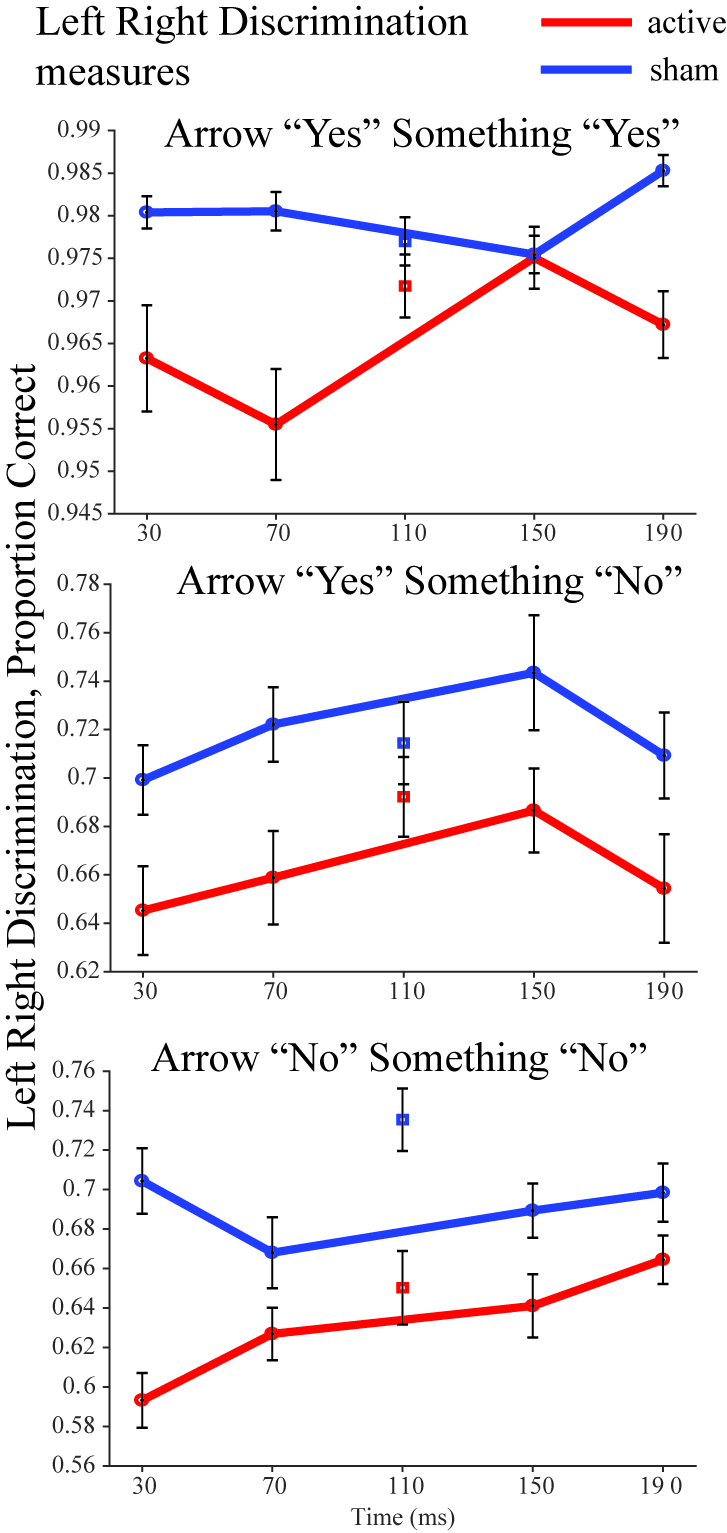


**Figure S6.** Left/Right discrimination capacity under all levels of reported awareness. Arrow and Something refer to detection questions. Arrow “No” and Something “No” is equivalent to the PcU measure. Plots conform to the same format as figure 2.

**Participant instructions**

You will be shown arrows (Figure. 2 below) that appear very briefly against background noise (Figure. 3 below). Sometimes the arrow will not appear. Your task is to decide if you saw the arrow and which direction it was pointing. You will be asked 3 questions about this:

Did you see the arrow? Yes/No.

Which direction was it pointing in? Left/Right.

Did you see something? Yes/No.

It is important for the purpose of this study that you give your best guess for the direction discrimination question (Left/Right?), even when you don’t see the arrow, i.e. do not simply press the same button when you think no stimulus has been presented.

Sometimes you will see something that might have been an arrow but you cannot be sure. This is why we ask the extra question: ‘Did you see something?’ If you have the impression that you saw something which might have been the arrow, but cannot be sure, you should say ‘Yes’ to this question and ‘No’ to the ‘Did you see the arrow?’ question. You should say ‘Yes’ to the arrow question if you were consciously aware of the arrow. Also, we sometimes show non-arrows (Figure 4). If you see this or think you might have, you should answer ‘Yes’ to the something question. If you saw nothing but the noise you should answer ‘No’ to both the arrow and the something questions.

The order of the questions will change from one testing session to the next. At the start of each session you will be given some practice trials to become familiar with the order of the question and refresh your memory of the task.

You will be asked 3 questions: i) ‘Was the arrow pointing left or right?’ which will appear on the screen as ‘L/R'. ii) ‘Did you see the arrow?’ which will appear as ‘arrow Y/N’. iii) ‘Did you see something?’ which will appear as ‘something Y/N’. Responses will be taken on the keyboard number pad using the 1,3,4 and 6 keys (see Figure 5). The buttons on the left (1 and 4) are for responding ‘Left’ and the ones on the right (2 and 6) are for ‘Right’. The upper buttons (4 and 6) are for responding ‘Yes’ and the lower ones (1 and 3) are for responding ‘No’. If at any point you enter the wrong response please tell the experimenter immediately so that they can make a note of it.

Sessions will involve approximately eight blocks of testing, each consisting of 80 trials. To complete the experiment, you will undergo 3 sessions over the course of 3 to 6 days, plus calibration days.

Calibration days will involve establishing thresholds for the task. This means we will vary the ease of the task so that we can get a stable level of performance, which we will then use in subsequent sessions. This will involve short blocks of trials. There will also be one of these shorter blocks at the start of each normal testing session to check that levels of performance are constant, and adjust them if necessary.

During the first few days we will be assessing your susceptibility to phosphenes. Phosphenes are apparent flashes of light caused by the TMS. The experimenter will go through this with you in more detail during the first session.

If you have any questions, please ask.

Thanks for reading this and participating in our experiments.


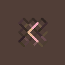

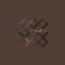

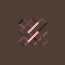
 Figure 2. Figure 3. Figure 4.

The arrow that you will The noise in the absence The non-arrow.

be looking for. of an arrow.

7

8

9

4

5

6

2

1

3

Yes

No

Right

Left

Figure 5.

The layout of the button responses on the number pad of the keyboard.

**References**

Corwin, J. (1994). On measuring discrimination and respones bias: unequal numbers of targets and distractors and two classes of distractors. *Neuropsychology*, *8*(1), 7.

Fleming, S. M. (2017). HMeta-d: hierarchical Bayesian estimation of metacognitive efficiency from confidence ratings. *Neuroscience of Consciousness*, *2017*(1), nix007.

Fleming, S. M., & Lau, H. C. (2014). How to measure metacognition . *Frontiers in Human Neuroscience* .

Green, D. M., & Swets, J. A. (1966). *Signal detection theory and psychophysics*. New York ; London: Wiley.

Lee, M. D. (2008). BayesSDT: software for Bayesian inference with signal detection theory. *Behavior Research Methods*, *40*(2), 450–456.

Lee, M. D., & Wagenmakers, E.-J. (2014). *Bayesian cognitive modeling: A practical course*. Cambridge university press.

Macmillan, N. A., Creelman, C. D., MacMillan C.D., N. A. & C., Macmillan, N. A., Creelman, C. D., & MacMillan C.D., N. A. & C. (1990). *Detection theory : a user’s guide*. Cambridge: Cambridge university press.

Sullivan, L. M., & D’Agostino, R. B. (1992). Robustness of the t test applied to data distorted from normality by floor effects. *J Dent Res*, *71*(12), 1938–1943.
