# Supplementary material for: Causal manipulation of feed-forward and recurrent processing differentially affects measures of consciousness
Source: Neurosci Conscious. 2020 Sep 7;2020(1):niaa015. doi: 10.1093/nc/niaa015 (PMC7475771; doi:10.1093/nc/niaa015)
Supplement: niaa015_Supplementary_Data [file niaa015_supplementary_data.zip › OPEN_SCIENCE_BADGE_APPLICATION_FORM_11Oct19.docx]

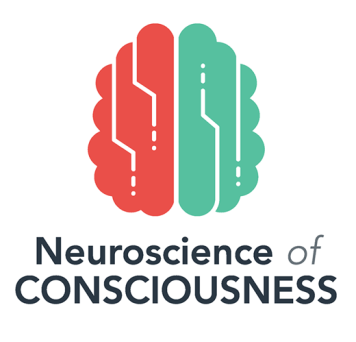
 **OPEN SCIENCE BADGE APPLICATION FORM**

**Open Data Badge**

Please provide the URL, DOI, or other permanent path for accessing the data in a public, open access repository.

I

<https://osf.io/dwfqv/>

Is there sufficient information for an independent researcher to reproduce the reported results? If no, explain.

Yes

**Open Materials Badge**

Please provide the URL, DOI, or other permanent path for accessing the materials in a public, open access repository.

I

<https://osf.io/dwfqv/>

Is there sufficient information for an independent researcher to reproduce the reported methodology? If no, explain.

Yes

**Preregistered Badge**

Please provide the URL, DOI, or other permanent path to the registration (and, if applicable, the analysis plan) in a public, open access repository.

I

<https://osf.io/v9nzi/>

Was the plan preregistered prior to the examination of the data or observing the outcomes? If no, explain.

Yes

Were there additional registrations for the study other than the one reported? If yes, provide links and explain.

Yes, an amendment – but also reported and pre-registered prior to the majority of data collection and all primary confirmatory analyses, see <https://osf.io/x9pig/>

For Preregistered and Analysis plan badge: were there any changes to the preregistered analysis plan for the primary confirmatory analysis? If yes, explain.

See above

For Preregistered and Analysis plan badge: are all of the analyses described in the registered plan reported in the article? If no, explain.

Yes
